# M2 Macrophage-Derived Small Extracellular Vesicles Ameliorate Pyroptosis and Intervertebral Disc Degeneration

**DOI:** 10.34133/bmr.0047

**Published:** 2024-07-01

**Authors:** Kaihui Zhang, Lilong Du, Zhenhua Li, Zhenxin Huo, Li Shen, Shan Gao, Yiming Jia, Meifeng Zhu, Baoshan Xu

**Affiliations:** ^1^ Department of Minimally Invasive Spine Surgery, Tianjin Hospital, Tianjin University, Tianjin 300211, China.; ^2^ First Teaching Hospital of Tianjin University of Traditional Chinese Medicine, Tianjin 300193, China.; ^3^ Department of Stomatology, Chifeng Municipal Hospital, Chifeng, Inner Mongolia 024000, China.; ^4^ College of Life Sciences, Key Laboratory of Bioactive Materials (Ministry of Education), Nankai University, Tianjin 300071, China.

## Abstract

Intervertebral discs (IVDs) have a limited self-regenerative capacity and current strategies for IVD regeneration are unsatisfactory. Recent studies showed that small extracellular vesicles derived from M2 macrophage cells (M2-sEVs) inhibited inflammation by delivery of various bioactive molecules to recipient cells, which indicated that M2-sEVs may offer a therapeutic strategy for the repair of IVDs. Herein, we investigated the roles and mechanisms of M2-sEVs on IVD regeneration. The in vitro results demonstrated that M2-sEVs inhibited pyroptosis, preserved cellular viability, and promoted migration of nucleus pulposus cells (NPCs). Bioinformatics analysis and verification experiments of microRNA (miR) expression showed that miR-221-3p was highly expressed in M2-sEVs. The mechanism of action was explored and indicated that M2-sEVs inhibited pyroptosis of NPCs through transfer of miR-221-3p, which suppressed the expression levels of phosphatase and tensin homolog and NOD-, LRR-, and pyrin domain-containing protein 3. Moreover, we fabricated decellularized ECM-hydrogel (dECM) for sustained release of M2-sEVs, which exhibited biocompatibility and controlled release properties. The in vivo results revealed that dECM-hydrogel containing M2-sEVs (dECM/M2-sEVs) delayed the degeneration of intervertebral disc degeneration (IDD) models. In addition to demonstrating a promising therapeutic for IDD, this study provided valuable data for furthering the understanding of the roles and mechanisms of M2-sEVs in IVD regeneration.

## Introduction

Low back pain (LBP) is a common health condition associated with high socioeconomic burden and its incidence is increasing rapidly with the aging population. Currently, over 80% of the world’s population are estimated to be afflicted by LBP in their lifetime, and approximately 40% of all cases of LBP are attributed to intervertebral disc degeneration (IDD) [1]. Current treatments are limited to pain relief by conservative therapies and invasive surgical procedures treated as last resorts [2,3]. Thus, there is an ever-increasing clinical requirement for new therapeutic strategies for IDD.

IDD is characterized by the loss of resident cells and extracellular matrix (ECM) integrity. In particular, the senescence and death of nucleus pulposus cells (NPCs) and catabolic remodeling of the ECM within the central interior of the intervertebral disc (IVD) are detrimental hallmarks in the progression of IDD. As degeneration proceeds, the cells undergo phenotypic changes and the production and aggregation of inflammatory cytokines further promotes ECM breakdown [4]. Hence, therapeutic treatment of impaired and dysfunctional NPCs represents an important target for attenuated IDD progression.

In recent years, emerging studies have shown the potential of using small extracellular vesicles (sEVs) as therapeutic and cell-alternative strategies for tissue regeneration [5]. Typically, sEVs have diameters ranging between 30 and 200 nm and operate to transport important substances for cell-to-cell communication [6,7]. Isolated sEVs have the advantages of ease of storage and avoidance of the disadvantages associated with cell transplantation, which include phenotypic alterations post-transplant and uncontrolled fate and degree of activity. In addition, sEVs carry various proteins, bioactive molecules, and RNAs, which regulate the activity of recipient cells [8]. The sEVs derived from M2 macrophages (M2-sEVs) have been shown to possess anti-inflammatory, pro-healing, and tissue repair functions, conveying similar actions to their parent M2 macrophage cells [9,10]. Therefore, exploiting the beneficial actions of M2-sEVs may hold promise for preventing IDD and stimulating regenerative processes. However, there have been few bodies of work that have focused on the roles of M2-sEVs on the repair of IVD and related mechanisms.

Inflammation plays an important role in IDD pathogenesis [11]. Pyroptosis is a form of regulated cell death initiated by inflammation, inflammasomes, and the activation of caspases [12,13]. Previous studies have indicated that inflammasome-mediated pyroptosis of NPCs plays a critical role in driving IDD [14,15]. During the progression of IDD, the gradually increasing activation of the NOD-, LRR-, and pyrin domain-containing protein 3 (NLRP3) inflammasome elicits the cleavage of caspase-1 and secretion of interleukin (IL)-1β, which results in cell rupture and damage to the surrounding ECM [14]. Therefore, we speculate that M2-sEVs may provide a protective effect against inflammasome activation and NPCs pyroptosis. Furthermore, the mechanism by which M2-sEVs may ameliorate NPCs pyroptosis is unclear. Multiple studies have shown that sEVs contain a variety of nucleic acid biomolecules, among which microRNA (miR) play important roles in regulating cellular metabolism and proliferation through suppressing the expression of target mRNA [16]. Previous studies have shown that miR-221-3p could suppress the expression of cyclin-dependent kinase inhibitor 1B (CDKN1B) directly [17]; Similarly, M2 macrophage-derived miR-221-3p plays an important role in osteosarcoma [18]. Therefore, it deserves further study.

Free sEVs in aqueous solution are difficult to retain and stabilize at the site of IDD, and are rapidly cleared and/or destroyed [19], which brings an additional challenge to treat IDD. Furthermore, it has proven difficult to achieve a long-term therapeutic effect from a single injection of sEVs [20], and repeated injections are not feasible options for the clinical treatment of IDD. To circumvent these issues, a carrier material that exhibits the characteristics of appropriate degradation rates and the controlled and sustained release of loaded sEVs is ideal [21,22]. Recently, tissue-specific ECM materials realized following decellularization have been shown to possess good biocompatibility without immunogenicity and have been widely implemented for the repair of multiple tissue types [23]. In hydrogel form, decellularized ECM-hydrogels provide a cell-supporting niche that mimics the native ECM, and these have been used as cell and bioactive factor carriers, demonstrating sustained-release properties and the treatment of degenerative diseases [24]. Therefore, we hypothesize that the combination of M2-sEVs with decellularized nucleus pulposus ECM-hydrogel carriers (dECM/M2-sEVs) could enhance the reparative effect in IDD, compared to M2-sEVs alone.

**Fig. 1.**
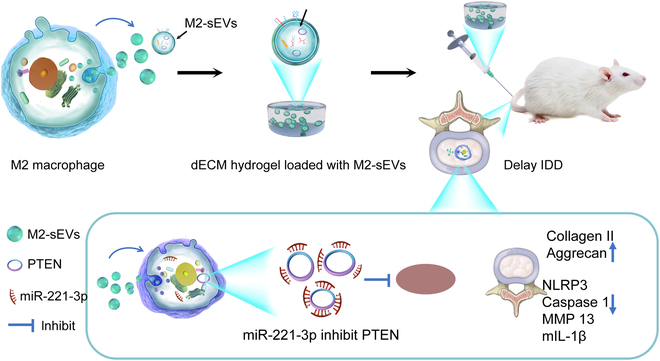
Schematic diagram for M2 macrophage-derived sEVs release from dECM-hydrogels and the subsequent delivery of miR-221-3p to inhibit nucleus pulposus cell pyroptosis via modulation of the PTEN/NLRP3 signaling pathway.

This study aimed to explore the effects and mechanisms of M2-sEVs on NPCs pyroptosis, and the therapeutic effect of dECM/M2-sEVs in the prevention and repair of IDD in vivo using murine models. We further explored the mechanisms of action using bioinformatic analysis and verification experiments and determination of miR content in M2-sEVs. We next determined the effects of M2-sEVs delivery of miR to NPCs and downstream regulation of pyroptosis, the inflammatory response, and the phosphatase and tensin homolog (PTEN)/NLRP3 signaling pathway. Moreover, we evaluated the propensity of dECM/M2-sEVs to promote ECM regeneration in IDD models in vivo. Overall, our investigation revealed the therapeutic effects and mechanisms of M2-sEVs in IDD, providing new insights and promise for realizing a new direction for IDD treatment (Fig. 1).

## Materials and Methods

### Patient sample collection and analysis

Human degenerative nucleus pulposus (NP) tissues were obtained from patients who underwent lumbar surgery owing to chronic LBP. According to the Pfirrmann classification [25], 12 patients with IDD (ages 40 to 65 years) were selected and classified into grade II, III, IV, or V based on T2-weighted sagittal images and then the expression of Caspase-1 protein was detected to indicate degenerative NP. All experimental protocols in this study were approved by the ethics committee of Tianjin Hospital and the patients provided signed informed consent forms (2020012). Briefly, the NP tissues were fixed in 4% paraformaldehyde for 24 h, before rinsing with distilled water 3 times. The samples were embedded in paraffin and cut into 5-μm sections, deparaffinized in xylene, rehydrated through a graded series of ethanol, and rinsed in distilled water. The endogenous peroxidase activity was blocked with 3% hydrogen peroxide, and the sections were incubated with 3% bovine serum albumin (BSA) for 30 min at room temperature. Sections were incubated with the primary antibody, anti-Caspase-1 (1:150, Proteintech, Cat# 22915-1-AP), overnight at 4 °C. Goat anti-rabbit IgG-HRP secondary antibody (1:50, Proteintech, Cat# SA00001-2) was incubated with sections at room temperature for 50 min. Then, the sections were stained with DAB for 1 min and with hematoxylin for 3 min. A standard light microscope and camera were used to visualize and capture the observations.

### NPCs culture and H_2_O_2_ treatments

Human NPCs were purchased from Sciencell (Catalog Number: 4800) and cultured as previously reported [26]. Briefly, NPCs were cultured in DMEM/F12 (HyClone, UK) in the presence of 10% fetal bovine serum (FBS), 100 U/ml penicillin, and 100 U/ml streptomycin in a humidified incubator (5% CO_2_; 37 °C). To generate a model of degenerating NPCs, the cells were treated with different concentrations hydrogen peroxide (H_2_O_2_).

### Preparation of M2 macrophages

Human monocytic THP-1 cells were obtained from the Cell Resource Center (Peking Union Medical College; 1101HUM-PUMC000057). THP-1 cells were cultured in Roswell Park Memorial Institute (RPMI-1640) medium (Cell Resource Center, Peking Union Medical College, CCCM019) containing 10% FBS (Gibco) and supplemented with 25 mM HEPES. THP-1 cells were differentiated into resting-state macrophages by incubation with 100 ng/ml phorbol 12-myristate 13-acetate (Sigma, P1585) for 24 h. Then, M2 macrophage polarization was induced by incubation with 20 ng/ml of IL-4 (Peprotech, #200-04) and 20 ng/ml of IL-13 (Peprotech, #200-13). The identification of M2 macrophage was confirmed using flow cytometry for markers, including CD68, CD163, and CD206 (Fig. S1).

### Isolation and characterization of M2-sEVs

Exosome-depleted FBS was prepared in advance, as previously reported [27]. After 72 h of culture, the supernatants of M2 macrophages were collected and centrifuged at 2,000×*g* for 10 min to remove cells, and then at 10,000×*g* (4 °C) for 20 min to remove larger vesicles. The supernatant was filtered through 0.22-μm pore size syringe filters before ultracentrifugation at 120,000×*g* (4 °C) for 2 h. The isolated sEVs were stored at –80 °C until further use. The morphology and size distribution of the collected sEVs were observed using a transmission electron microscope (TEM; Hitachi HT7700, Tokyo, Japan) and the size distribution of sEVs was measured using a Nanosizer (Malvern, UK). In addition, the collected sEVs were identified by their expression of the surface markers, CD81, TSG101, and Calnexin of cells marker, using Western blotting and flow cytometry to identify CD9, CD63, and CD81. The uptake of sEVs by NPCs was also studied by labeling M2 macrophage-derived sEVs (M2-sEVs) with the PKH-26 kit (Sigma-Aldrich, USA, MINI26), according to the manufacturer’s instructions. Briefly, M2-sEVs were incubated with 10 μl of PKH-26 dye and 500 μl of Dilution C at room temperature for 30 min in the dark. Then, 5% BSA was used to stop the staining process. The labeled M2-sEVs were centrifuged at 120,000×*g* for 2 h, and then the M2-sEVs were resuspended in an appropriate volume of pre-cooled phosphate buffer saline (PBS). Labeled M2-sEVs were incubated with NPCs cultured in confocal dishes for 6, 12, 24, and 48 h. Subsequently, cells were fixed with 4% paraformaldehyde and then stained with 4′,6-diamidino-2-phenylindole (DAPI) for 2 min. Images were viewed and captured using a confocal fluorescence microscope.

### Cell viability assay and Transwell migration assay

Cell Counting Kit-8 (CCK-8, Biosharp) assays were performed to estimate cell viability. A total number of 5×10^3^ NPCs per well were seeded into 96-well plates. The NPCs were treated with different concentrations of H_2_O_2_ (100, 200, 300, 400, 500, and 600 μM), and cell viability was detected according to the CCK-8 protocol. Then, the effects of different concentrations of M2-sEVs (0.2, 2, and 20 μg/ml) on the viability of NPCs were also measured by CCK-8. The optical density (O.D.) values were measured using a microplate reader (Thermo Fisher, USA) at 450 nm. Transwell migration assays were used to determine the effect of M2-sEVs on the migration of NPCs, as previously described [28]. Briefly, the NPCs were adjusted to a density of 3 × 10^3^ cells/well and seeded in the upper chamber of Transwell plates, while M2-sEVs (concentrations: 0, 0.2, 2, and 20 μg/ml) were added to the lower chamber with serum-free medium. After incubation for 24 h, cells on the upper surface of the Transwell filters were swabbed off, and the migrated NPCs on the bottom of the filters were fixed by 4% paraformaldehyde and then stained with 0.1% crystal violet for 10 min. Finally, the migrated NPCs were observed under a microscope and counting across 5 randomly captured fields of view.

### TUNEL, Hoechst33342/PI, and Annexin V/PI staining

Terminal deoxynucleotidyl transferase-mediated dUTP-biotin nick end labeling (TUNEL) staining kit (Beyotime Biotechnology, Shanghai, China, #C1088) was used to determine the pyroptosis rates of NPCs, following the manufacturer’s instructions. Briefly, NPCs were incubated with H_2_O_2_ for 2 h, and then the culture medium was replaced by culture medium containing M2-sEVs. After 24 h of co-culture, the NPCs were fixed with 4% paraformaldehyde and permeabilized with 0.3% Triton X-100 for 5 min. After being washed with PBS, the samples were incubated with the TUNEL stain. The prepared cells were analyzed using a fluorescence microscope and the positive cells were quantified by ImageJ (National Institutes of Health, USA). Hoechst33342/propidium iodide (PI) double fluorescence staining was used to further determine pyroptosis of NPCs, as previously reported [29]. Pyroptotic cells exhibited low blue/high red light. Briefly, pretreated NPCs in 6-well plates were gently washed with PBS before Hoechst33342 and PI mixed staining solution was added to the plates, incubated for 30 min at 4 °C, and observed under a fluorescence microscope. The Annexin V/PI double-standard staining was used to examine PI-positive cells by flow cytometry (BD Biosciences, USA). Briefly, 5 × 10^5^ of the pretreated NP cells were collected and resuspended with 100 μl of binding buffer. Annexin V (5 μl) and 5 μl of PI working solution were added to each tube and incubated for 30 min in the dark. The rate of positive cells was detected by flow cytometry.

### Detection of reactive oxygen species

The level of reactive oxygen species (ROS) in NPCs was measured by the ROS detection kit (Solarbio, China, #D6470). Briefly, the pretreated NPCs were incubated with 10 μM 2,7-dichlorodi-hydrofluorescein diacetate (DCFH-DA) at 37 °C for 30 min in the dark. After incubation, the cells were washed with serum-free medium 1 to 2 times. The intracellular ROS level in NPCs was observed under fluorescence microscopy and the positive cells were quantified by ImageJ (NIH).

### Enzyme-linked immunosorbent assay

The supernatant of cultured NPCs after different treatments were collected and the concentrations of IL-1β were measured using the human IL-1β ELISA (enzyme-linked immunosorbent assay) Kit or the IL-18 ELISA Kit, following the manufacturer’s instructions. Briefly, 50 μl of different concentrations of standards and samples was added to the ELISA plates; then, 100 μl of enzyme labeling reagent was added and incubated at 37 °C for 60 min in the dark. Next, the reaction solution was removed and the chromogenic reagent was added and incubated at 37 °C for 15 min. Finally, 50 μl of stop solution was added to stop the reaction process and the OD values of each well were measured using a multiwell plate reader at 450 nm.

### Western blot analysis

Total protein was extracted from NPCs or NP tissues, according to the previously described protocols [30]. Briefly, total protein concentration was detected by bicinchoninic acid assay (BCA, Solarbio, China, #PC0020). Then, the lysates of NPCs or NP tissues were diluted at a ratio of 1:5 with 5× loading buffer and heated at 95 °C for 5 min. The protein mixture was loaded into the wells of SDS-PAGE gels for electrophoresis at 120 V for 1 h before transfer onto a polyvinylidene fluoride (PVDF) membrane (Merck-Millipore) for 60 min at 300 mA. After blocking for 3 h with 5% non-fat dried milk in TBST, the PVDF membranes were incubated with primary antibodies at 4 °C overnight. Following 3 thorough washes with TBST, horseradish peroxidase (HRP)-labeled secondary antibodies were incubated with the membranes at 37 °C for 1 h. ECL reagent was added to the membranes and blots were detected using an Amersham Imager 600. The primary antibodies and dilutions were as follows: anti-Caspase-1(p20) (Proteintech, China, 22915-1, 1:1000), anti-NLRP3 (Proteintech, China, 19771-1, 1:1000), anti-mIL-1β (Abcam, UK, ab283818, 1:1,000), anti-MMP13 (Proteintech, China, 18165-1, 1:1,000), anti-Aggrecan (Abcam, UK, ab36861, 1:1,000), anti-Collagen II (Abcam, UK, ab188570, 1:1,000), and anti-PTEN (CST, USA, 9559S, 1:1,000).

### Quantitative real-time PCR analysis

Reverse transcriptase quantitative real-time polymerase chain reaction (RT-qPCR) was performed as previously reported [30]. Briefly, total RNA was extracted with Trizol reagent (Invitrogen), and the PrimeScript RT reagent kit (TaKaRa, Japan) was used for reverse transcription of the purified RNA. RT-qPCR analysis was performed using the SYBR Premix Ex Taq II kit (TaKaRa, Japan) and detected on a Roche LightCycler 480 sequence detection system. GAPDH was used as a control gene. The 2^−ΔΔCt^ method was conducted to calculate the relative expression of each gene. Cycle threshold (Ct) was defined as the amplification cycle when real-time fluorescence intensity of the reaction reached the set threshold. RT-qPCR primer sequences are provided in Table S1.

### IDD miRNA array data acquisition and bioinformatics analysis

Non-coding RNA profiling array data obtained from IDD tissue samples were downloaded from the Gene Expression Omnibus (GEO) database of National Center for Biotechnology Information (NCBI). The keyword used to search for the data was “intervertebral disc degeneration” and the GSE116726 dataset [31] was acquired. A total of 3 NP tissues from IDD patients and 3 normal NP tissues from patients with traumatic lumbar fractures were included in this dataset. The R project for statistical computation was used to filter, normalize, and standardize the original expression data. The screening criteria were log2 FC≥1 and *P* < 0.05. The selected differentially expressed miRNAs were processed by Shengxinren web tool to chart a Volcano plot of the expression data. Previous studies of M2-sEVs content indicated miR-221-3p as an abundant miRNA [17]. The expression of miR-221-3p was found to be down-regulated in the GSE116726 IDD dataset. Target genes of miR-221-3p were predicted across 4 databases (starBase, mirDIP, miRTarbase, and miRmap) and the common targets were identified. Protein interaction analyses were conducted between the predicted target genes on the STRING website, and Cytoscape software was used to identify key functional proteins for investigation.

### RNA fluorescence in situ hybridization

The cytoplasmic localization of M2-sEVs released miR in relation to the nuclei and mRNA of NPCs was identified using RNA fluorescence in situ hybridization (FISH) technology, as previously reported [30]. The nucleic acid probe based on the sequence of miR-221-3p (5′-GAAACCCAGCAGACAATGTAGCT-3′) was labeled by green fluorescence (Alexa 488), whereas red fluorescence (Alexa 594) indicated the presence of PTEN mRNA. DAPI was used to designate cell nucleus. RNA FISH was then conducted according to the instructions of the FISH Detection Kit (QIAGEN). Imaging was acquired with a confocal fluorescence microscope.

### Dual-luciferase reporter assay

Dual-luciferase reporter assays were carried out according to previously reported protocols [32]. In short, the 5′-flanking sequence of the PTEN promoter was inserted into a pGL3 vector. Then, the pGL3-PTEN vector with 40 ng of pRL-TK vector was transfected into NPCs using Lipofectamine 2000. The NP cells were co-transfected with the indicated RNA oligonucleotides and reporter plasmids including pmir-report-PTEN-WT and pmir-report-PTEN-Mut. According to the manufacturer’s instructions, dual-luciferase activity was measured after 48 h using the Dual-Glo Luciferase System Kit (Beyotime Biotechnology, China, #RG027).

### Preparation of dECM-hydrogel

Fresh bovine spine was purchased from a local slaughterhouse, and fresh NP tissues were carefully dissected from the thoracolumbar segment. The NP tissues were minced and soaked in 75% ethanol for 6 h, washed with sterile distilled water 5 times, and then submerged in sterile 2% SDS solution and placed on a 100 rpm shaker for 72 h. The 2% SDS solution was changed every 8 h. The SDS was washed continuously with sterile distilled water until the solution no longer foamed. Then, the tissue was eluted with sterile DNase I/RNase A (Solarbio, China, #D8071/R8021) solution for 48 h. The tissue was then repeatedly washed with sterile distilled water before being vacuum freeze-dried for 48 h. The freeze-dried tissue was broken into pieces using a commercially available mechanical pulverizer, and the resultant crushed decellularized ECM was sealed and stored at −20 °C until further use. The decellularized ECM (and natural NP tissue as a comparison) was fixed in OCT and cut to a thickness of 5 μm. Sections were washed for 5 min before staining with hematoxylin and eosin (H&E; Solarbio, China, G1120) and Alcian Blue (Solarbio, China, G2541). Alternatively, sections were fixed in 4% paraformaldehyde and permeabilized with 0.1% Triton X-100 before DAPI (Sigma) staining for 5 min and observation by fluorescence microscopy (Leica, German).

The prepared dECM powder of NP tissue was placed in ethylene oxide for sterilization. Concentrations (0.5%, 1%, and 2%) of dECM thermosensitive hydrogels were prepared based on the protocols of a previous study [33]. A total mass of 60 mg of sterilized dECM, 10 ml of 0.01 mol/L HCl, and pepsin (1/10 the mass of dECM powder; concentration, 0.5% mg/ml) were mixed and stirred. When the dECM powder was completely digested into a liquid state, 1 ml of 0.1 mol/L pre-cooled NaOH was pH adjusted to neutral, then 1 ml of pre-cooled 10×PBS was added before the mixture was placed in a 37 °C incubator for 30 min until gelation. The same procedure was used to prepare 1% and 2% mg/ml dECM thermosensitive hydrogels by adjusting the quantity of dECM powder, accordingly. The hydrogel states were then photographed after tube inversion.

### Rheology characterization of the dECM-hydrogel

After preparing the corresponding concentrations of dECM thermosensitive hydrogels on ice, a small amount of hydrogel was quickly dropped onto the rheology instrument (Brookfield, American), according to the manufacturer’s suggested protocols. The temperature was set to increase from 15 °C to 40 °C and the rheology of the hydrogel was recorded.

### Cell viability and morphology assessments

The viability of NPCs grown on dECM-hydrogels was evaluated by Live/Dead staining (YUHENG, Suzhou, China, L6037/YP0059) after 1-, 4-, and 7-day culture. Briefly, in 24-well plates, 100 μl of NPCs was seeded onto pre-planted dECM-hydrogels and cultured in 500 μl of complete culture medium. Firstly, the medium was removed and washed with PBS. Secondly, the stain working solution of Calcein-AM (live cell) and PI (dead cell) was added and kept in darkness at 37 °C for 15 to 20 min. Then, staining results were observed by a laser confocal microscope (Olympus Corporation, Japan).

To assess cell morphology and cytoskeletal distribution, fluorescein isothiocyanate (FITC)-phalloidin staining was also conducted after 1-, 4-, and 7-day culture of cells on dECM-hydrogels. Briefly, the dECM-hydrogel with NPCs was fixed and permeabilized with 0.1% Triton X-100. The samples were then incubated in 1% BSA blocking solution before cellular actin was stained with FITC-phalloidin for 2 h and the nuclei were stained with DAPI for 2 min. The final images were captured using a confocal fluorescence microscope.

### The release profile of M2-sEVs from the dECM-hydrogel and effects on NPCs

The M2-sEVs release profile from dECM-hydrogels was determined by CD81 ELISA Kit (MEIMIAN, Shanghai, China). Briefly, 450 μl of dECM-hydrogel (1.33%) and 150 μl of M2-sEVs (1μg/μl) were mixed in PBS on ice to prepare a 1% dECM/M2-sEVs system, which was placed in an incubator at 37 °C for 30 min until gelation. Subsequently, 200 μl of PBS was added and replaced with fresh PBS every 2 days. The supernatant was collected every 2 days until day 30, free sEVs were detected using ELISAs, and the percentage of released M2-sEVs was calculated. To detect the effect of M2-sEVs release from dECM-hydrogel on NPCs, the viability and proliferation of H_2_O_2_-treated cells cultured with dECM-hydrogel or dECM/M2-sEVs for 1, 4, and 7 days were examined using CCK-8 assays. The NPCs grown with hydrogel complexes without H_2_O_2_ were used as control samples. The O.D. values were measured using a microplate reader at 450 nm.

### Animal models and surgical procedures

All experimental and animal care procedures were approved by the Animal Research Ethics Committee of Tianjin Nankai Hospital (No. 2021-031). Twenty-five 12-week-old male Sprague-Dawley (SD, SCXK-2019-0010) rats weighing 300 ± 20 g were used for animal models. Rat tail needle puncture-induced IDD models were generated as previously described [34]. In brief, the rats were anesthetized by isoflurane. The intervertebral spaces of coccygeal discs (Co) were exposed (Co6/7, Co7/8, Co8/9, Co9/10, and Co10/11 were operated). To induce IDD in rat caudal spine, an 18-gauge syringe needle was inserted 5 mm into each disc, rotated 90° clockwise, and held in position for 45 s [34]. Magnetic resonance imaging (MRI) was used to determine whether the models were successful in inducing IDD. The rats were randomly divided into 5 groups: (a) dECM/M2-sEVs; (b) M2-sEVs; (c) control (no needle puncture); (d) dECM-hydrogel only; (e) and PBS group (treated with PBS). Following the successful generation of IDD models, 4 μl of material (or PBS) was injected through a micro-syringe (18-gauge needle) into the NP tissue, and was repeated 4 weeks later. X-rays and MRI were performed at 4 and 8 weeks postoperatively and histological analysis was evaluated at 8 weeks postoperatively.

### Radiographic evaluation (x-rays and MRI analysis)

At 4 and 8 weeks post-operation, the rats were anesthetized and radiographed using an x-ray system. Radiographs were obtained using an exposure time of 30 s and a penetration power of 35 kV. The change in IVD height was then quantified using the intervertebral disc height index (DHI) according to previous reports. The differences in DHI of discs were calculated as percentages by using the following calculation: (%DHI = post-operative DHI/pre-operative DHI ×100).

T2-weighted midsagittal sections were obtained using a 3.0-T MRI scanner. The rat was fixed onto a small-animal MRI coil. The region of the rat tail was used as a region of interest for scanning. The MRI scan parameters were as follows: spin echo repetition time, 2,275 ms; echo time, 80 ms; number of excitations, 8; field of view, 5 cm; slice thickness, 1.5 mm. The Pfirrmann MRI grading score was used to record the results.

### Histological analysis

The coccygeal discs of rats were fixed with paraformaldehyde for 72 h and transferred to decalcify in EDTA solution for 2 weeks. After decalcification was complete, the samples were embedded in paraffin and cut into 5-μm sections, deparaffinized in xylene, rehydrated through a graded series of ethanol, and rinsed in distilled water. H&E staining, Safranine-O-Fast Green staining, and Alcian Blue staining were performed for histological observations in each group. The histological scores were evaluated based on the modified histologic grading system developed by Ji et al.[31]. In brief, the cellularity and morphology of the AF, NP, and the border between the 2 structures were examined.

### Statistical analysis

The data are shown as means ± standard deviation (SD). For normally distributed data, Student’s *t* tests were used to determine statistically significant differences between 2 independent groups of data; otherwise, the nonparametric Kruskal–Wallis test was applied. Means of multiple groups were compared by one-way analysis of variance (ANOVA) with Fisher’s least significant difference (LSD). Statistical analysis was conducted using SPSS v20.0 (IBM Corp., Armonk, NY, USA). *P* values < 0.05 were considered statistically significant.

## Results

### Confirmation of Caspase-1 expression in human degenerative NP

**Fig. 2.**
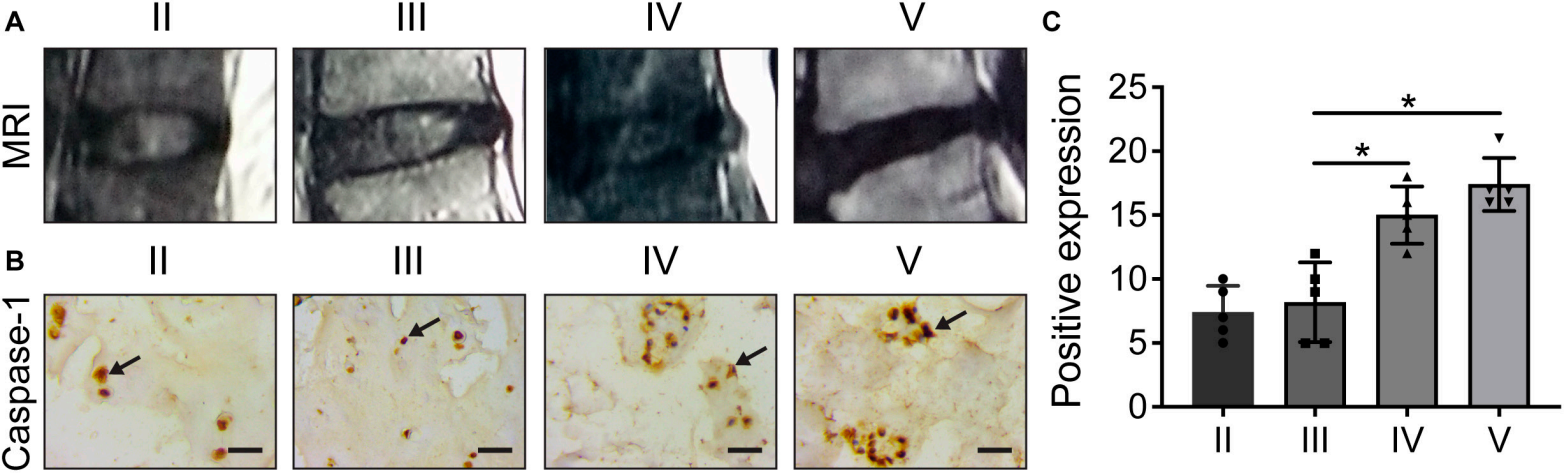
The expression of Caspase-1 increases with the degree of IDD. (A) Typical MRI images of II to V Pfirrmann grading. (B) Representative photographs of IHC for Caspase-1-positive cells in degenerated NP tissue (scale bar: 50 μm). (C) Corresponding quantification of positive protein expression levels. Data are presented as the mean ± SD for each group (*P < 0.05).

According to the Pfirrmann grading after T2-weighted MRI of midsagittal sections (Fig. 2A), grades II to V of IDD NP tissue was selected and IHC was carried out to observe the presence and expression of Caspase-1 protein, which is a key protein of pyroptosis [29]. The results showed that Caspase-1 was expressed in degenerated NP tissue and the expression of Caspase-1 increased with the degree of IDD (Fig. 2B). These results suggested that pyroptosis levels were likely to be increased in degenerative NP tissues.

### Characterization of M2-sEVs

**Fig. 3.**
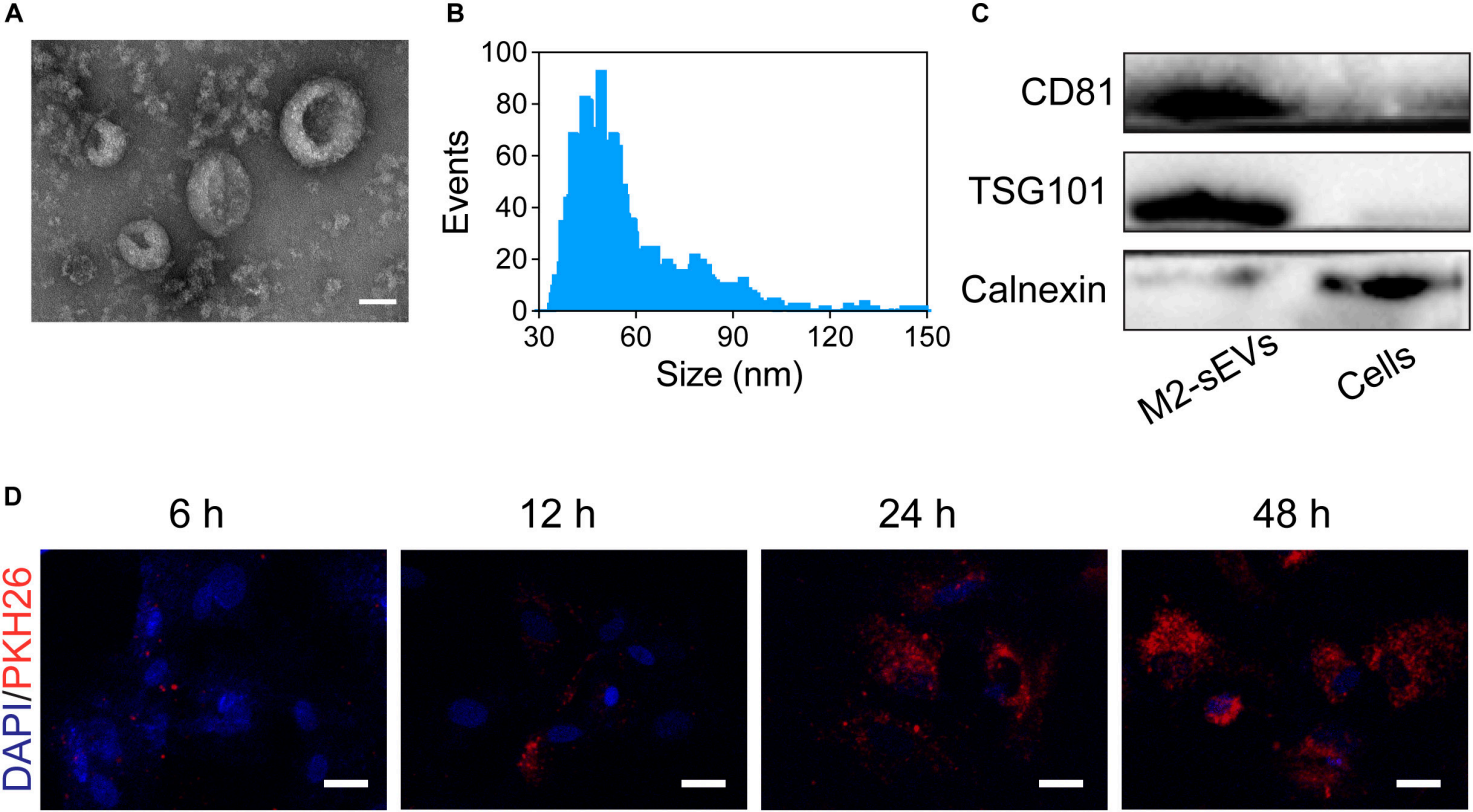
Identification of M2-macrophage-derived sEVs (M2-sEVs). (A) Morphology of M2-sEVs identified by TEM. Scale bar: 50 nm. (B) Particle size distribution of M2-sEVs measured by NanoSizer. (C) Western blotting analysis of the surface biomarkers CD81, TSG101, and Calnexin of M2-sEVs and cells. (D) The time-dependent cell internalization ability of M2-sEVs within NPCs (scale bars: 20 μm).

In order to confirm the successful isolation of M2-sEVs, TEM, dynamic light scattering (DLS) by NanoSizer, and Western blot analyses were performed. TEM showed that M2-sEVs were round in shape and had the typically cup-like lipid membrane structure with sizes ranging between 30 and 150 nm (Fig. 3A). NanoSizer measurements showed that the approximate size of M2-sEVs varied between 30 and 150 nm, with the most frequent diameter sizes being between 45 and 60 nm (Fig. 3B). Western blotting showed that M2-sEVs were positive for the characteristic sEV surface markers, including CD81 and TSG101, but not Calnexin of cells marker (Fig. 3C). The macrophage-secreted small extracellular vesicle expression of CD81, CD9, and CD63 was identified by a flow cytometry experiment (Fig. S2). In addition, M2-sEVs were labeled with PKH26, and the PKH26-labeled M2-sEVs were identified within NPCs following co-incubation at 6, 12, 24, and 48 h, indicating that NPCs readily took up M2-sEVs and reached saturation at 24 h in vitro (Fig. 3D).

### Effects of M2-sEVs on NPCs treated with H_2_O_2_ in vitro

**Fig. 4.**
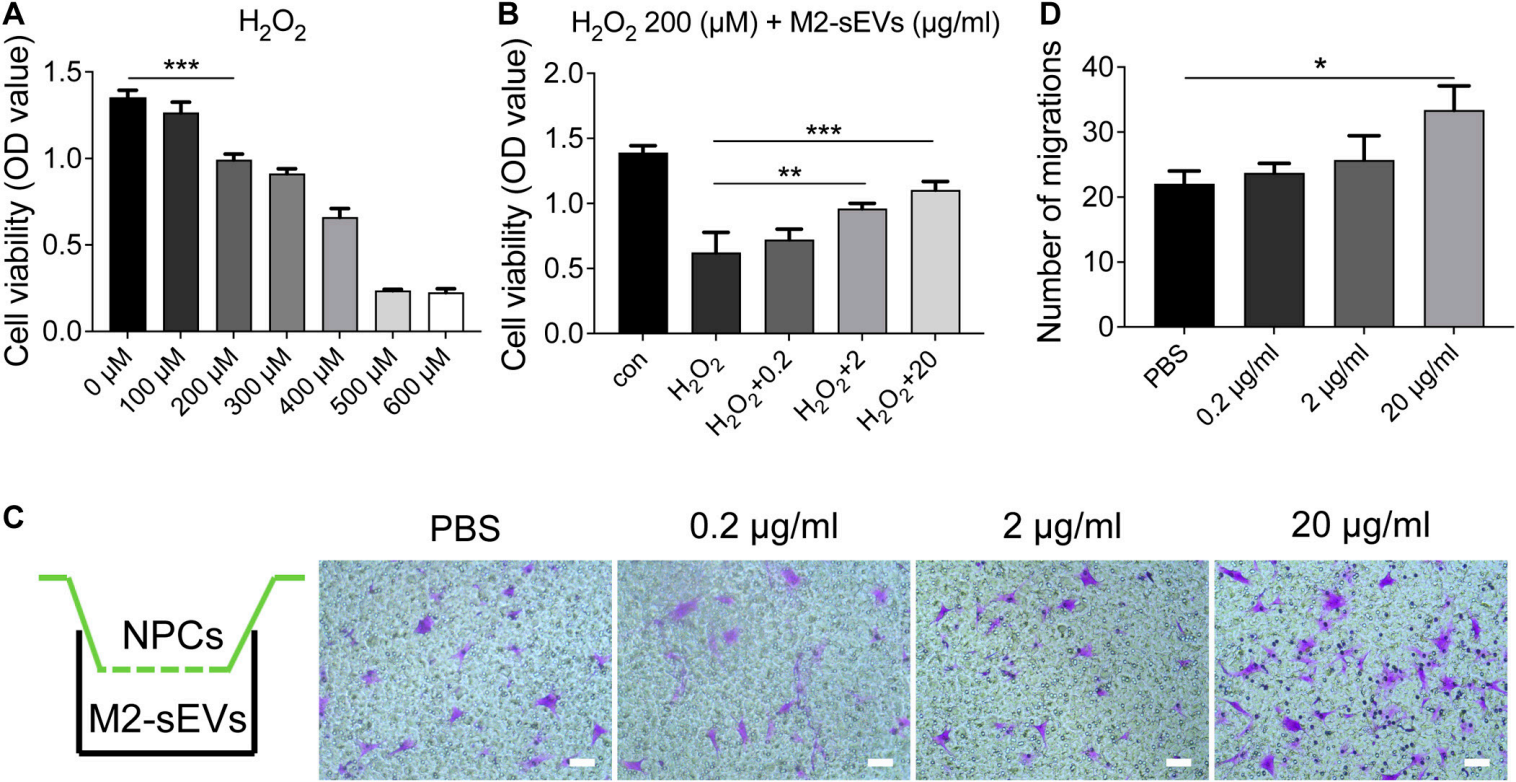
The viability and migration of NP cells treated by H_2_O_2_ and M2-sEVs. (A) CCK-8 assay showed that the cell viability of NPCs decreased with increasing H_2_O_2_ concentrations. (B) CCK-8 assays showed that the viability of H_2_O_2_-treated NPCs was significantly rescued by the addition of M2-sEVs. (C) Transwell migration assays showed that the migration of NPCs increased with the increase of M2-sEVs concentration; Scale bars: 50 μm. (D) Quantitative analysis of the number of migrating cells. Data are shown as the mean ± SD (*P < 0.05*,* **P < 0.01, ***P < 0.001).

In order to study the effect of M2-sEVs on NPC models of oxidative stress under a H_2_O_2_ microenvironment, NPCs were cultured in conditioned medium containing different concentrations of H_2_O_2_ and M2-sEVs. The CCK-8 assay results showed that the cell viability of NPCs decreased with increasing H_2_O_2_ concentrations. When the concentration of H_2_O_2_ exceeded 200 μM, the cell viability decreased significantly (*P* < 0.05) (Fig. 4A); thus, 200 μM H_2_O_2_ was selected for oxidative stress injury models, which was consistent with previous studies [29]. Next, NPCs were treated with 200 μM H_2_O_2_ for 2 h before the culture medium was changed and different concentrations of M2-sEVs (0.2, 2, or 20 μg/ml) were incubated with NPCs for 24 h. The CCK-8 assays showed that the viability of H_2_O_2_-treated NPCs was significantly rescued by the addition of M2-sEVs and the cell survival rate increased in an M2-sEVs concentration-dependent manner (Fig. 4B). These data indicated that M2-sEVs could significantly attenuate H_2_O_2_-mediated NPCs death. In order to verify the effect of M2-sEVs on the migration of NPCs, Transwell assays were performed and showed that the migration of NPCs increased with the increase of M2-sEVs concentration (Fig. 4C). The number of migrated NPCs was significantly increased in the 20 μg/ml M2-sEVs-treated groups (PBS vs. 20 μg/ml, *P* = 0.007) (Fig. 4D). Therefore, 20 μg/ml M2-sEVs were selected for subsequent experiments.

### M2-sEVs inhibits H_2_O_2_-induced pyroptosis of NPCs

**Fig. 5.**
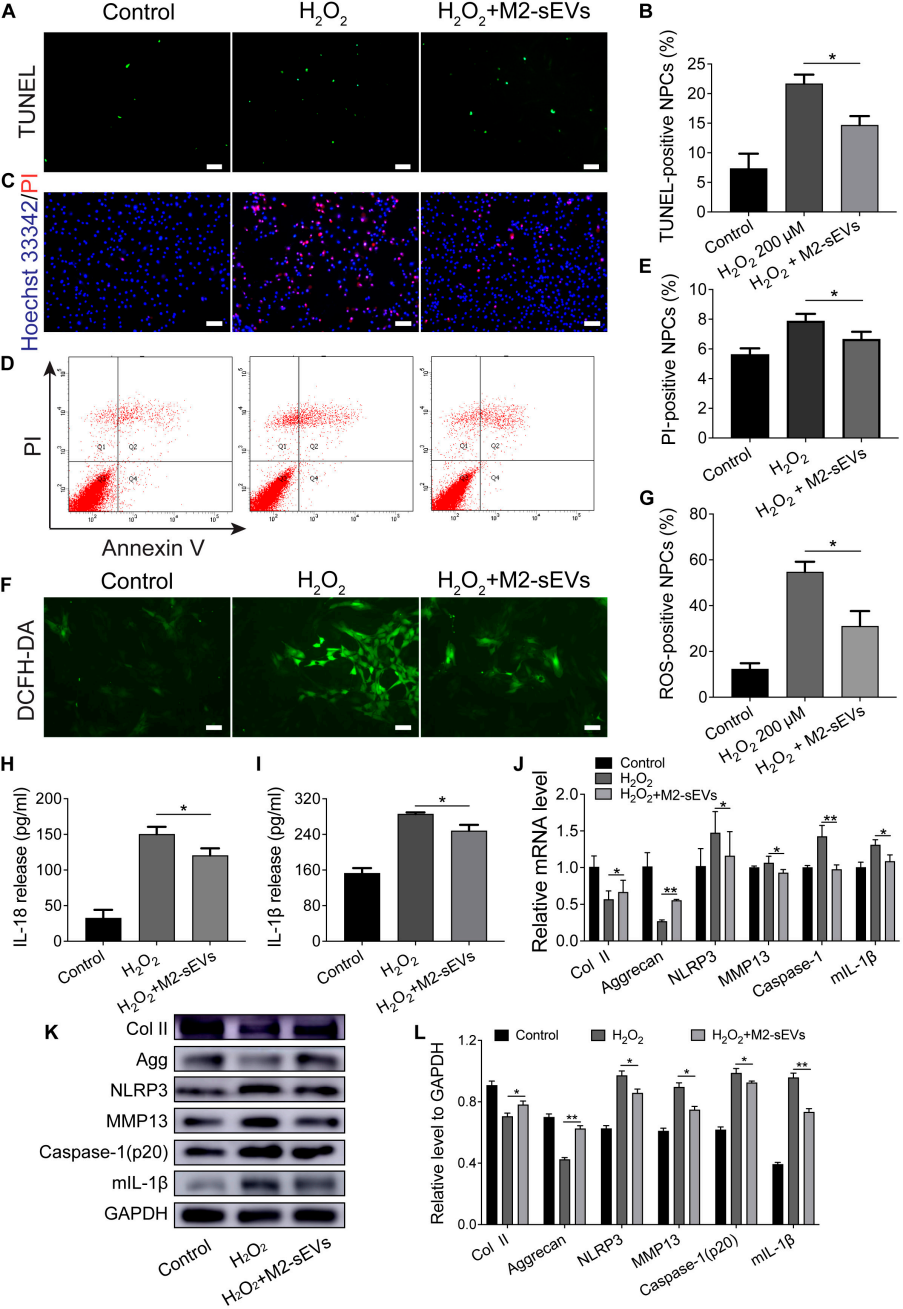
M2-sEVs inhibits H_2_O_2_-induced pyroptosis of NP cells. (A and B) The TUNEL staining and quantitative analysis was used to determine the pyroptosis of NPCs treated with H_2_O_2_ (200 μM) with or without M2-sEVs (scale bar: 100 μm). (C) Hoechst33342/PI double fluorescence staining was used to detect the changes of cell membrane permeability after NPCs was treated with H_2_O_2_ with or without M2-sEVs (scale bar: 100 μm). (D) Annexin V/PI flow cytometry staining was used to detect PI-positive cells in NPC treated with H_2_O_2_ with or without M2-sEVs and quantitative analysis was shown in (E). (F) DCFH-DA staining was used to detect the changes of oxygen content after NPCs was treated with H_2_O_2_ with or without M2-sEVs (scale bar: 100 μm). (G) Quantitative analysis of ROS-positive NPCs. The secretion of (H) IL-18 and (I) IL-1β from NPCs treated with H_2_O_2_ with or without M2-sEVs, as detected by ELISA. The relative expression levels of Col II, Agg, NLRP3, MMP13, Caspase-1 (p20), and mIL-1β were assessed by (J) RT-qPCR and (K) Western blotting in HNPC treated with H_2_O_2_ with or without M2-sEVs. (L) Quantitative analysis of Western blot protein bands. Data are presented as the mean ± SD for each group (*P < 0.05, **P < 0.01).

Subsequently, we assessed whether the protective effects of M2-sEVs were effective against H_2_O_2_-induced pyroptosis. TUNEL staining results showed that the pyroptosis of NPCs significantly decreased in the H_2_O_2_+M2-sEVs group when compared with the H_2_O_2_-treated group (Fig. 5A and B). These results were verified by Hoechst33342/PI immunofluorescence staining (Fig. 5C), which were consistently similar to the results of Annexin V/PI flow cytometry analysis (Fig. 5D and E). DCFH-DA staining and quantitative analysis showed that M2-sEVs decreased oxidative stress levels and intracellular ROS production, compared with the H_2_O_2_-treated group (Fig. 5F and G). In addition, ELISA results showed that the H_2_O_2_+M2-sEVs groups showed a decrease in the production of secreted IL-18 and IL-1β, compared to the H_2_O_2_-treated group (Fig. 5H and I). RT-qPCR quantitative analysis indicated that H_2_O_2_ treatments significantly decreased the expression of collagen II (Col II) and aggrecan, but increased the expression of NLRP3, MMP13, Caspase-1 (p20), and mIL-1β in NPCs. However, this phenomenon was reversed after co-culturing with M2-sEVs (Fig. 5J). Consistent with these data, Western blots and quantitative results confirmed that total protein expression followed a similar trend to the mRNA expression (Fig. 5K and L). Overall, these results implied that M2-sEVs alleviated H_2_O_2_-induced inflammatory responses and pyroptosis of NPCs.

### M2-sEVs-encapsulated miR-221-3p inhibits NPC pyroptosis via regulating the PTEN/NLRP3 signaling pathway

**Fig. 6.**
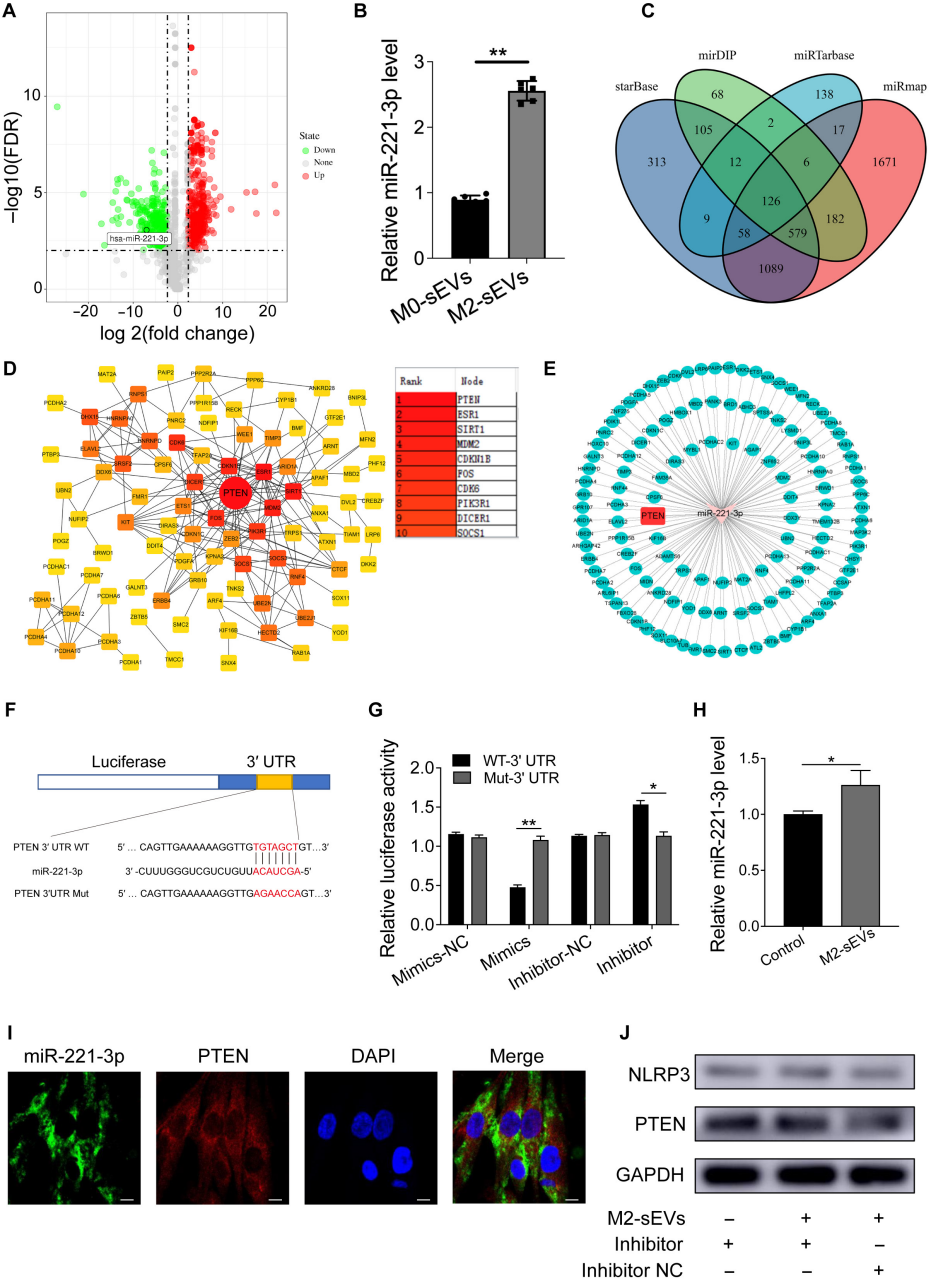
M2-sEVs regulates pyroptosis of NPCs through modulation of the miR-221-3p/PTEN/NLRP3 pathway. (A) Volcano plot of differentially expressed miRNA in the GSE116726 dataset; the expression of has-miR-221-3p was significantly down-regulated. (B) RT-qPCR was performed to detect the level of miR-221-3p in M0-sEVs and M2-sEVs. (C) Venn diagram of miR-221-3p predicted mRNA targets indicated by 4 databases. (D) PPI analysis was used to identify key regulating proteins. (E) Construction of the miR-221-3p predictive regulation network to determine putative miR–mRNA interactions. (F) The putative binding site between miR-221-3p within the 3′ UTR of PTEN mRNA and design of mutated binding site. (G) Dual-luciferase reporter assay was performed to verify the specific interaction between miR-221-3p and WT-PTEN but not Mut-PTEN; (H) RT-qPCR was performed to detect the level of miR-221-3p in NPCs co-cultured with PBS or M2-sEVs. (I) RNA FISH was performed to detect the cellular localization of M2-sEVs-delivered miR-221-3p and NPC PTEN mRNA (scale bar: 10 μm). (J) Western blotting was used to detect the effect of M2-sEVs-delivered miR-221-3p on the protein expression of PTEN and NLRP3. Data are shown as the mean ± SD for each group (*P < 0.05, **P < 0.01).

To understand the possible mechanism of the protective effects of M2-sEVs on NPCs, we performed bioinformatics analyses on publicly available database entries. Analysis of the original data obtained from the GSE116726 non-coding RNA array dataset indicated that significantly and differentially expressed miR species were evident in IDD patients (Fig. 6A). The data revealed that the expression of miR-221-3p was significantly down-regulated in the dataset. Furthermore, the expression level of miR-221-3p was subsequently examined in the M0-sEVs and M2-sEVs, and the results showed that the expression of miR-221-3p was higher in M2-sEVs, compared to sEV derived from control macrophages (M0-sEVs) (Fig. 6B). Thus, our data indicated that up-regulated miR-221-3p could play a potentially positive role in mitigating IDD.

By searching the miR databases (starBase, mirDIP, miRTarbase, and miRmap), the mRNA targets of miR-221-3p were predicted. A total of 126 putative targets of miR-221-3p were predicted across the 4 databases (Fig. 6C). Subsequently, the predicted genes were input into the STRING website, and the results were then introduced into Cytoscape. The protein–protein interaction (PPI) network suggested that PTEN was the common interacting protein with a potential critical regulator role (Fig. 6D). At the same time, Cytoscape was used to display the miRNA–mRNA predictive regulatory network (Fig. 6E). The binding site of miR-221-3p to the 3′ UTR of PTEN mRNA was predicted by the PicTar database, and a mutation sequence to negate binding site was designed (Fig. 6F). The binding site of miR-221-3p to PTEN mRNA was verified by dual-luciferase reporter assay (Fig. 6G). The results showed that compared with the mimic negative control group, the miR-221-3p mimics significantly reduced the fluorescent intensity of the WT-PTEN group but not the Mut-PTEN group, and introduction of a miR-221-3p inhibitor significantly rescued the fluorescent intensity produced by the WT-PTEN group, whereas the Mut-PTEN readings remained unchanged (Fig. 6G). Also, the cellular localization relationship between miR-221-3p and PTEN mRNA was verified by RNA FISH. The results showed that both miR-221-3p and PTEN mRNA were primarily co-located in the cytoplasm (Fig. 6I). Together, these data suggested that M2-sEVs deliver miR-221-3p that binds and regulates the expression of PTEN mRNA in NPCs.

Subsequently, we studied the changes of miR-221-3p expression levels in NPCs treated with M2-sEVs or PBS. The results showed that the expression level of miR-221-3p was significantly up-regulated in M2-sEVs-treated groups (Fig. 6H). Western blotting showed that NPCs incubated with M2-sEVs had inhibited the production of both PTEN and NLRP3, while co-treatment with miR-221-3p inhibitor reestablished the production of PTEN and NLRP3 proteins (Fig. 6J).

### Construction and characterization of thermosensitive dECM-hydrogel and loading and release of M2-sEVs

**Fig. 7.**
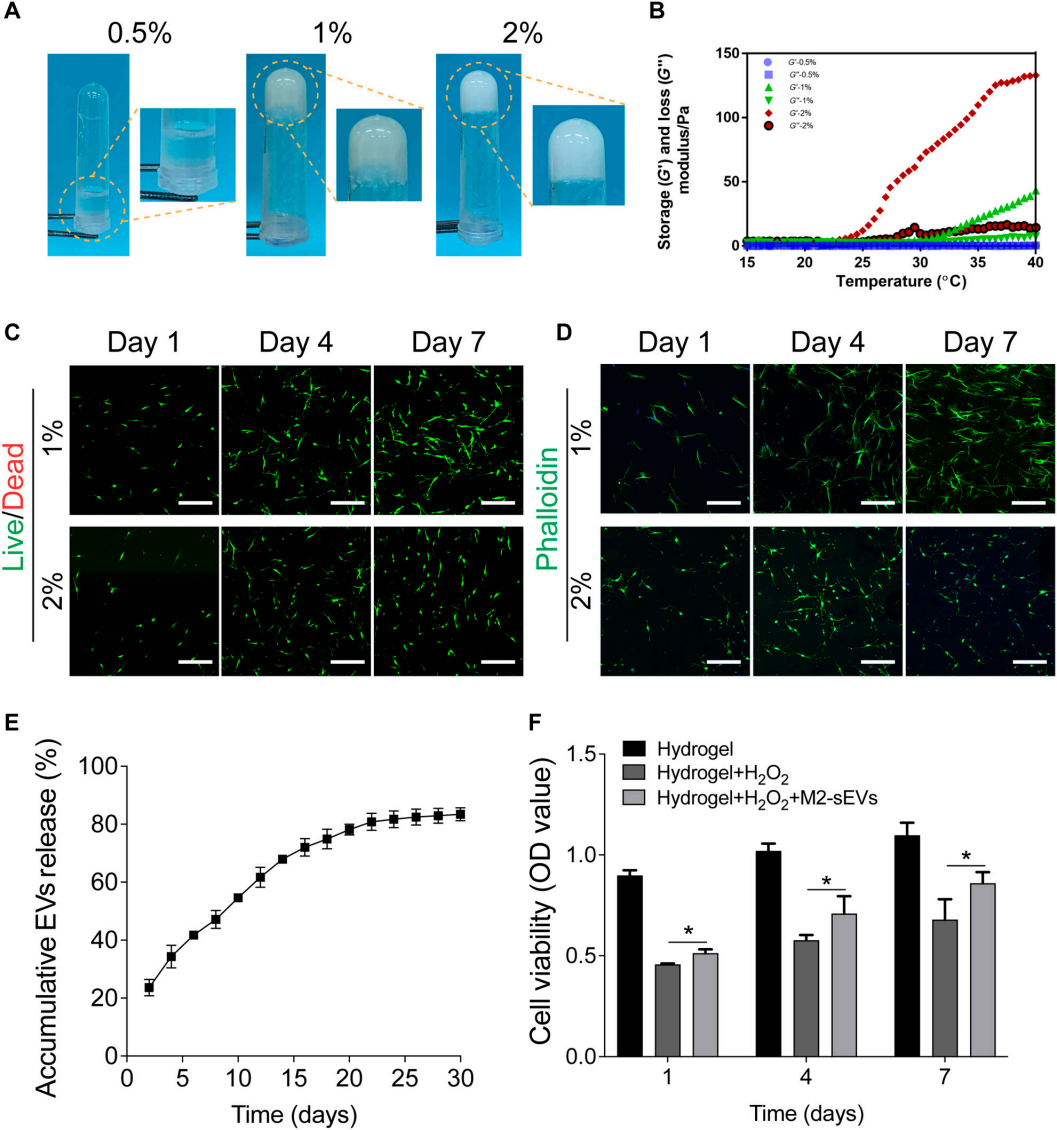
The characterization, biocompatibility, and releases of ECM hydrogel. (A) Photographs of dECM-hydrogels and their ability to form gels at different concentrations. (B) Rheological test results of dECM-hydrogels of different concentrations. (C) Live/Dead staining of NPCs seeded on 1% and 2% dECM-hydrogels. Scale bar: 100 μm. (D) Phalloidin staining of NPCs seeded on 1% and 2% hydrogel. Scale bar: 100 μm. (E) The sustained-release curves of M2-sEVs from 1% dECM-hydrogels. (F) CCK-8 assay results showed that the cell viability of H_2_O_2_-treated cells was rescued by M2-sEVs release from dECM-hydrogels. Data are shown as the mean ± SD for each group (*P < 0.05).

After the decellularization of fresh NP tissue, H&E showed almost complete removal of the cell nuclei and a loose ECM structure in dECM. DAPI staining indicated that most of the cellular nucleic acid content had been removed. Alcian Blue staining showed that the ECM, despite its looser connectivity, exhibited no obvious differences in glycosaminoglycan (GAG) content before or after decellularization treatment (Fig. S3). The gelation dECM solution showed that 0.5% dECM solution did not undergo gelation, whereas 1% and 2% solutions formed gelatinous substances. After gelation, the dECM solution formed adhesive, semi-transparent, and stable hydrogels (Fig. 7A). The rheological testing showed that the storage modulus (*G*′) and loss modulus (*G*″) of 0.5% dECM solution were lower, and the *G*′ values were not greater than the *G*″ values, which indicated that 0.5% dECM solution was unable to undergo gelation over a range of temperatures. The moduli of 1% and 2% dECM solutions increased gradually with the increase of temperature, and the *G*′ values were significantly higher than the *G*″ values at 37 °C, suggesting a higher viscosity compared with low viscosity at 15 to 25 °C (Fig. 7B). NPCs were seeded onto thermosensitive dECM-hydrogel to detect the biocompatibility of dECM-hydrogels. Live/Dead staining showed that majority of the NPCs seeded in 1% and 2% dECM-hydrogel were alive (green) and few cells had died (red), suggesting that the hydrogels derived from dECM had good biocompatibility (Fig. 7C). At the same time, phalloidin staining demonstrated that compared with 1% hydrogels, the morphology of NPCs grown on 2% hydrogels exhibited rounded morphologies and less cell spreading, which suggested that 2% dECM-hydrogels would not be conducive to supporting optimal NPC growth, whereas 1% dECM-hydrogels facilitated cell survivability, adhesion, and spreading (Fig. 7D).

We next assessed the sustained-release dynamics of M2-sEVs from dECM/M2-sEVs systems. The release curves indicated that the dECM-hydrogels were capable of prolonged and sustained-release of M2-sEVs. Over a period of 30 days, the dECM/M2-sEVs system progressively released the majority of M2-sEVs (83.47% ± 2.25%) (Fig. 7E). The CCK-8 assays revealed that cell viability of the H_2_O_2_-treated cells was significantly rescued by M2-sEVs release from dECM/M2-sEVs hydrogel systems when compared to the dECM-hydrogels without M2-sEVs (Fig. 7F).

### dECM/M2-sEVs alleviate IDD by inhibiting pyroptosis in vivo

**Fig. 8.**
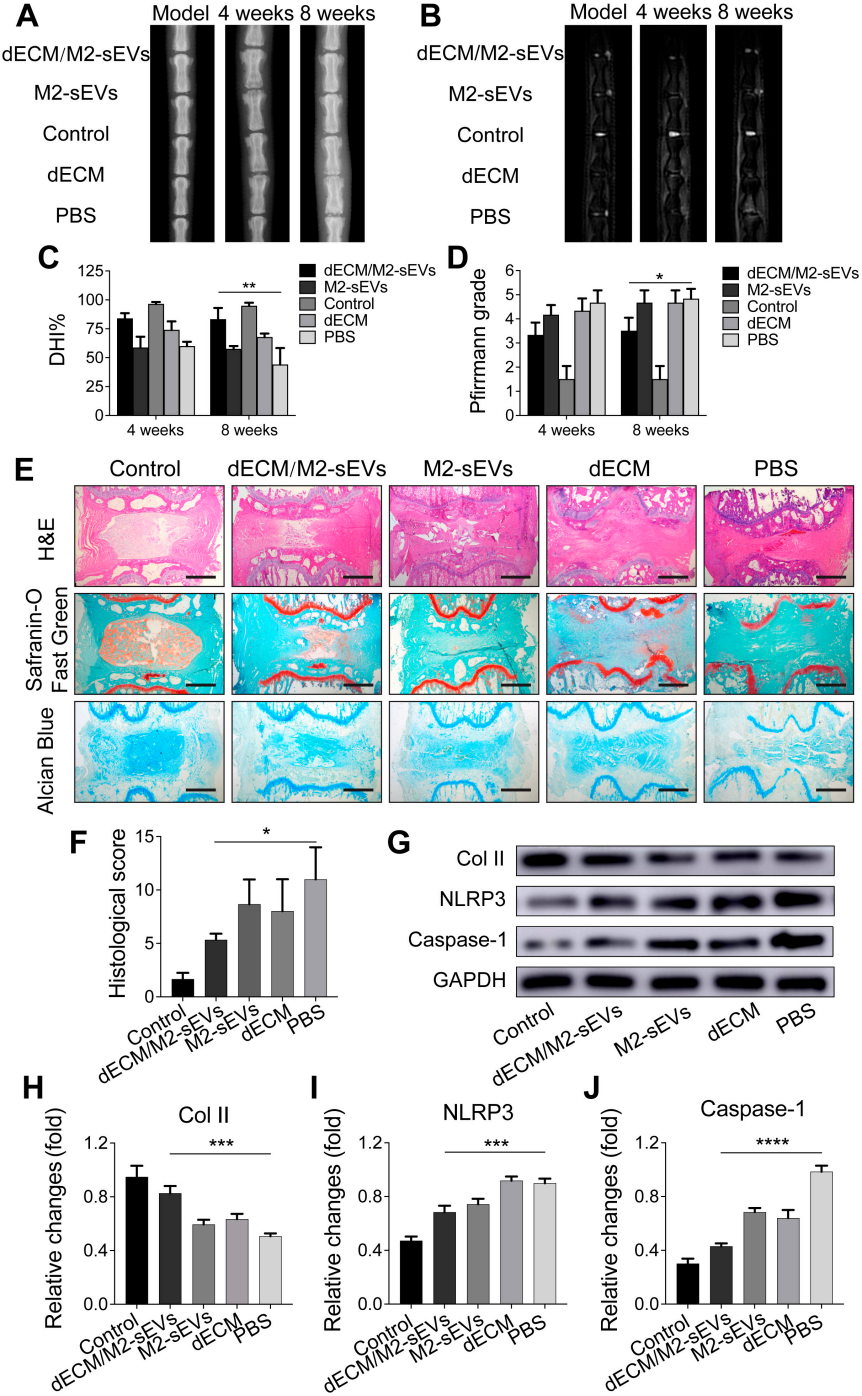
Radiography and histological evaluation of ECM hydrogel released M2-sEVs in the rat model. (A) X-ray images of the IVD at 0, 4, and 8 weeks. (B) Representative MRI images of rat coccygeal in the different groups. (C) DHI% of IVD differences among the groups at 4 and 8 weeks. (D) The Pfirrmann grading of the MRI images. (E) Images of H&E, Safranin-O-Fast Green, and Alcian Blue staining in each group (scale bar: 1 mm) and (F) histological scoring shown alongside. (G) Western blots and corresponding quantification of (H) Col II, (I) NLRP3, and (J) Caspase-1(p20) total protein expression in each group. Data are shown as the mean ± SD for each group (*P < 0.05, **P < 0.01, ***P < 0.001, ****P< 0.0001).

The therapeutic effects of our developed dECM/M2-sEVs system were evaluated in puncture-induced rat coccygeal IDD models. After 4 or 8 weeks of surgery, x-rays and MRI scans were used to evaluate the degree of rat coccygeal IDD (Fig. 8A to D). Based on the x-ray and MRI analysis, the height of the IVD in the PBS group showed a significant reduction compared to the control group (*P* < 0.05), and the dECM group and M2-sEVs group also showed different degrees of reduction. In contrast, the dECM/M2-sEVs group exhibited a degree of disc compression but had retained disc height, compared to the other groups. T2-MRI images illustrated that the NP integrity and water content were better preserved in the dECM/M2-sEVs group than in the other groups. Consistent with these results, Pfirrmann grading also revealed that the dECM/M2-sEVs group had the best reparative effects among all the treatment groups. Histological sections were obtained at 8-weeks post-operation. H&E, Saffron O-Fast Green, and Alcian Blue staining showed that the number of NPCs had decreased and the arrangement of ECM was disordered in the PBS group. The damage to the tissue structure was partially alleviated in the dECM-hydrogel and M2-sEVs groups, but the dECM/M2-sEVs group displayed a superior therapeutic effect on IDD, compared with the other treatment groups (Fig. 8E). Western blotting and RT-qPCR quantitative results showed that the expression of Caspase-1 and NLRP3 had significantly decreased, whereas the expression of Collagen II had significantly increased in the dECM/M2-sEVs group, compared to the PBS group. The other treatment groups did not present statistically significant differences to the PBS group (Fig. 8F to J).

## Discussion

IDD is the main cause of many spinal related diseases and morbidity, and is of substantial socioeconomic concern. The process of IDD progression is complex and multifactorial. In recent years, studies have shown that the increase in oxidative stress and subsequent inflammatory responses lead to cell death, and the destruction of the ECM in IVD tissues plays key determining roles in driving IDD pathogenesis. These pathophysiological cascades present as a hostile tissue microenvironment that poses a major challenge for therapeutic intervention, including cell-based therapies. Therefore, inhibiting the overactive inflammatory response and alleviating oxidative stress could be a holistic therapeutic approach to preventing ECM degradation and favoring IVD regeneration.

sEVs have been recognized as a promising alternative to cell-based strategies for achieving tissue regeneration. Macrophages have essential roles in both tissue repair and inflammation. The function of macrophages depends significantly on their polarization state, principally the pro-inflammatory M1 state or the anti-inflammatory M2 state. Recent studies have shown that M2 macrophage-derived sEVs (M2-sEVs) have broad application prospects in tissue repair and regeneration. Yan et al. [35] found that vascular smooth muscle cells co-cultured with M2 macrophages exhibited stronger dedifferentiation and vascular tissue repair ability. A recent study suggested that M2-sEVs serve as promising anti-inflammatory agents for rheumatoid arthritis treatment [9]. In the present study, the M2-sEVs were extracted by differential centrifugation. The M2-sEVs were disc-shaped with particle diameter sizes between 30 and 150 nm and presented sEV markers CD81, TSG101, and Alix, which were consistent with findings in multiple reports in the literature. Our results indicated that M2-sEVs rescued the viability of H_2_O_2_-treated NPCs and promoted the migration of NPCs.

Various types of programmed cell death contribute to the pathogenesis of IDD, including cell apoptosis, pyroptosis, and necroptosis. Pyroptosis is a form of regulated cell death initiated by inflammasomes and caspase activation. Pyroptosis is initiated with the formation of membrane pores, resulting in the inflow of water molecules and ions, loss of membrane potential, and cell lysis, resulting in the release of inflammatory substances [36]. Previous studies have shown that cell pyroptosis occurs in many degenerative diseases [37]. Pyroptosis is characterized by the presence of NLRP3-related inflammasomes, which promote the activation of Caspase-1 and the release of inflammatory cytokines. Consistent with these indicators, in this study, we found that the expression of Caspase-1 gradually increased with the severity of NP degeneration, which suggested that cell pyroptosis was an active mechanism of cell death occurring during IDD. Our results demonstrated that H_2_O_2_-induced NPCs exhibited up-regulated secretion of inflammatory cytokines (IL-18 and IL-1β), ROS accumulation [38], and increased NLRP3 and Caspase-1 protein production, which, in the case of IDD, we speculated would cumulate in ECM degradation and NP tissue destruction. In addition, Dai et al. [39] found that miR-148a from M2-sEVs attenuated myocardial ischemia/reperfusion injury by down-regulating the expression of TXNIP protein and inhibiting the toll-like receptor-4 (TLR4)/NF-κB/NLRP3 pyroptosis signaling axis. Given that M2 macrophages elicit anti-inflammatory and tissue repair roles, we speculated that M2-sEVs would inhibit cell pyroptosis of NPCs. In this study, RT-qPCR and Western blot analysis suggested that the H_2_O_2_ treatment significantly decreased the expression level of collagen II and aggrecan by NPCs. Moreover, this phenomenon was reversible when NPCs were co-cultured with M2-sEVs. These results suggested that M2-sEVs alleviated H_2_O_2_-induced inflammatory responses while protecting against ECM degradation in NPC in vitro models of IDD.

Increasing evidence suggests that sEVs payloads, such as proteins and nucleic acids, play crucial roles in regulating the activities and functions of recipient cells. As an important component of sEVs, miR have received attention for their roles in the prevention and promotion of various diseases [40]. The bioinformatics analyses we performed based on IDD datasets and those performed previously in M2-sEVs suggested that miR-221-3p was a likely candidate for regulating inflammatory responses. Indeed, Yang et al. [41] reported that miR-221-3p alleviated cell apoptosis and inflammatory responses via targeting cyclin-dependent kinase inhibitor 1B (CDKN1B) in CSE-treated 16HBE cell models of chronic obstructive pulmonary disease (COPD). One study indicated that miR-221-3p was down-regulated in human cartilage tissues, and this correlated closely with the degeneration grade index. Up-regulation of miR-221-3p prevented IL-1β-induced degradation of cartilage ECM through modulating the SDF1/CXCR4 signaling pathway [42]. Furthermore, in previous work by Li and Tang [17], array data on M2-sEVs content revealed an abundance of miR-221-3p. Here, we scanned miR target databases and input the data into PPI analysis software, which subsequently suggested PTEN as a key regulatory protein and candidate mRNA targeted by miR-221-3p. Additionally, our RNA FISH results indicated that both miR-221-3p and PTEN co-localized to the cytoplasm, indicating that a direct interaction between the two was plausible. Further data obtained by dual-luciferase reporter assay and Western blotting confirmed the inhibitory action of miR-221-3p on PTEN expression. Studies by Li et al. [43] and Shi et al. [44] also confirmed the targeting of miR-221-3p to PTEN mRNA. Sun et al. [45] found that miR-221-3p enhanced the activity of Akt through its inhibition of PTEN, and the up-regulation of miR-221-3p improved angiogenesis, migration, and proliferation rates while protecting against the apoptosis of mesenchymal stem cells. In our study, RT-qPCR demonstrated that the expression of miR-221-3p in M2-sEVs was significantly higher than that in the M0-sEVs. We found that while M2-sEVs-derived miR-221-3p inhibited the expression of PTEN and NLRP3, by introducing an inhibitor of miR-221-3p, the expression levels of PTEN and NLRP3 were rescued. Taken together, our data demonstrated that M2-sEVs-derived miR-221-3p could serve as a mitigator of IDD by suppressing the expression of PTEN and NLRP3, thereby offering a credible solution to decreasing the activation of the PTEN/NLRP3/inflammasome signaling pathway.

Biomaterials for NP tissue regeneration have focused mainly on injectable synthetic or biologically based hydrogels, including non-degradable polyethylene glycol, polylactic acid-glycolic acid or poly(hydroxyethyl methacrylate), biodegradable hyaluronic acid, collagen, chitosan, gelatin, alginate, and so on [46,47]. Decellularized tissue-specific ECM (dECM) materials have received considerable attention in recent years due to their high biocompatibility, excellent tissue integration, and intrinsic bio-inductive factors that can induce tissue-specific cell differentiation. Wachs et al. [48] decellularized NP tissues harvested from pigs and demonstrated that the decellularization process was capable of removing over 96% of the original DNA content without significantly reducing the collagen content and ECM microstructure, which remained similar to that observed in natural NP tissue. Hydrogels derived from dECM can offer additional functionality, such as acting as physical carriers of bioactive agents, but also retain tissue specificity for regulating cell fate. Borrelli and Buckley [24] developed an injectable biomimetic material that combined dECM with chitosan for the repair of IVD. In the present study, fresh bovine NP tissue was selected to prepare dECM-hydrogels. According to our test results, almost all cell-related components in the dECM were removed, and the dECM preserved the original ECM structure. The resulting 1% dECM-hydrogels demonstrated good biocompatibility and thermosensitivity (gelation at 37 °C), resulting in appropriate injectability potentiation of NPC proliferation and growth.

Another advantage of utilizing hydrogel carriers is that they facilitated the loading of M2-sEVs to realize the prolonged, sustained, and localized release of M2-sEVs at the injury site. Our results showed that the dECM/M2-sEVs maintained the release of M2-sEVs over 30 days, achieving a release of approximately 83.47%. Additionally, the rate of M2-sEVs was demonstrated to be sufficient for the significant rescue of cell viability of H_2_O_2_-treated NPCs. Thus, this study indicated that the dECM/M2-sEVs could achieve a dampening effect on the hostile, ROS-abundant and inflammatory microenvironment while providing biochemical cues similar to native NP tissue to promote cell proliferation.

Acellular scaffolds and sEV therapies are two emerging developmental directions in the field of IVD and IDD treatment. The combination of dECM and M2-sEVs can act together to enhance their tolerance to the hostile microenvironment in the early stage of degeneration. Recent studies indicated that the dECM-hydrogels combined with adipose-derived mesenchymal stem cell sEVs effectively modulated the microenvironment of IDD [49]. Hence, we further verified the regulatory effect of hydrogel sustained-release sEVs, and using M2-sEVs for a prominent anti-inflammatory and anti-pyroptotic effect on in vivo models of IDD. According to the results of our radiographic analysis and histological analysis, dECM/M2-sEVs effectively and synergistically treated IDD in contrast to the relatively weak therapeutic effects elicited by the injection of dECM or M2-sEVs alone.

In conclusion, a thermosensitive injectable dECM-hydrogel that incorporated M2-sEVs was developed for IDD therapy in this study. The dECM-hydrogel facilitated the encapsulation and sustained release of M2-sEVs while providing biochemical cues similar to native NP tissue to promote cell proliferation and IVD repair. Our results demonstrated that M2-sEVs delivery of miR-221-3p inhibited NPCs pyroptosis and dampened inflammatory responses via regulating the PTEN/NLRP3 pathway, resulting in the slowed progression of IDD. Moreover, our dECM/M2-sEVs system conveyed the dual actions of protecting NPCs from pyroptosis and promoting ECM regeneration after IDD in vivo. Our investigation revealed the beneficial therapeutic effects and mechanisms that can be achieved by M2-sEVs in IDD, providing new insights and promising routes toward innovative clinical treatments of IDD.

## Data Availability

The data used and/or analyzed in the current study are available on reasonable request.
