# Supplementary material for: M2 Macrophage-Derived Small Extracellular Vesicles Ameliorate Pyroptosis and Intervertebral Disc Degeneration
Source: Biomater Res. 2024 Jul 1;28:0047. doi: 10.34133/bmr.0047 (PMC11214826; doi:10.34133/bmr.0047)
Supplement: Supplementary 1 — Figs. S1 to S3 Table S1 [file bmr.0047.f1.docx]

**Supplementary** **Materials**


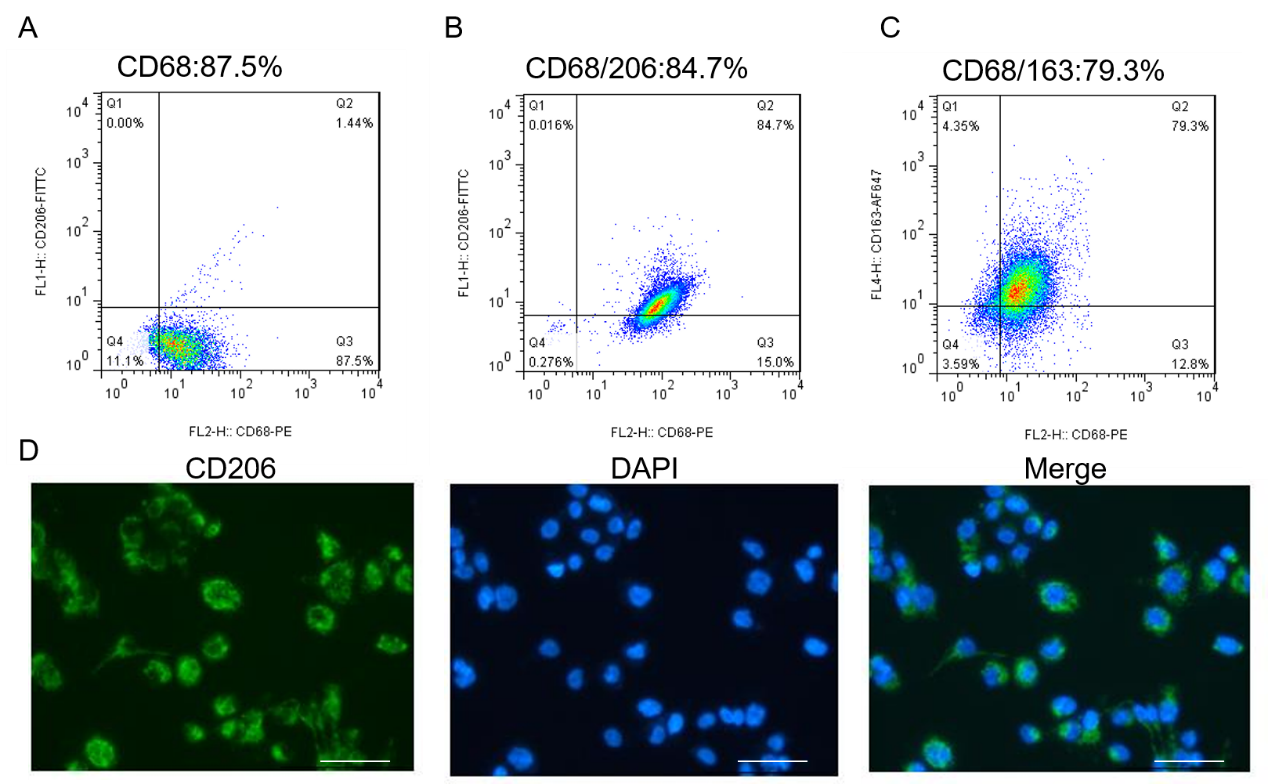


**Supplementary Fig. S1.** Identification of M2 macrophages by flow cytometry and immunofluorescence. (A-C) The identification of M2 macrophage markers including CD68, CD163 and CD206 by using flow cytometry; (D) The representative CD206 were detected by the immunofluorescence. Scale bar = 50 μm


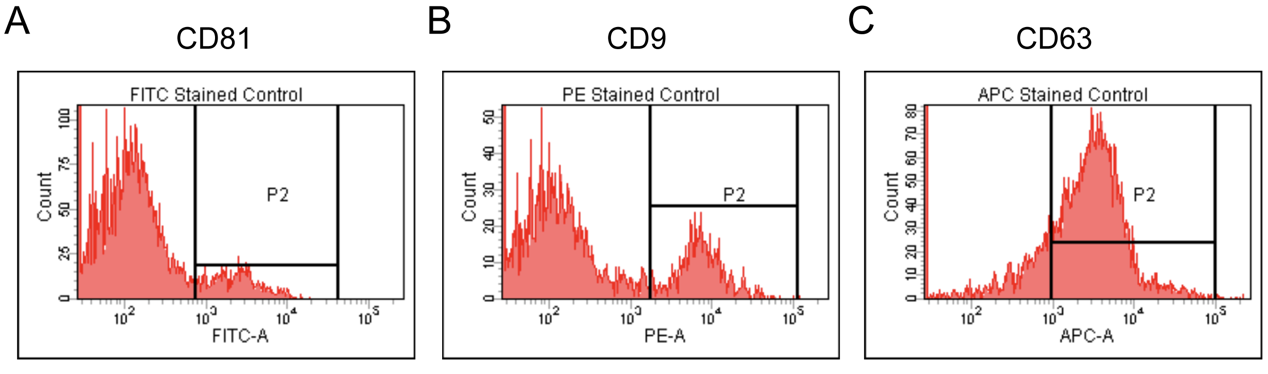


**Supplementary Fig. S2.** Identification of M2 macrophage derived sEV by flow cytometry. (A-C) The identification of M2 macrophage derived sEV markers including CD81, CD9 and CD63 by using flow cytometry.


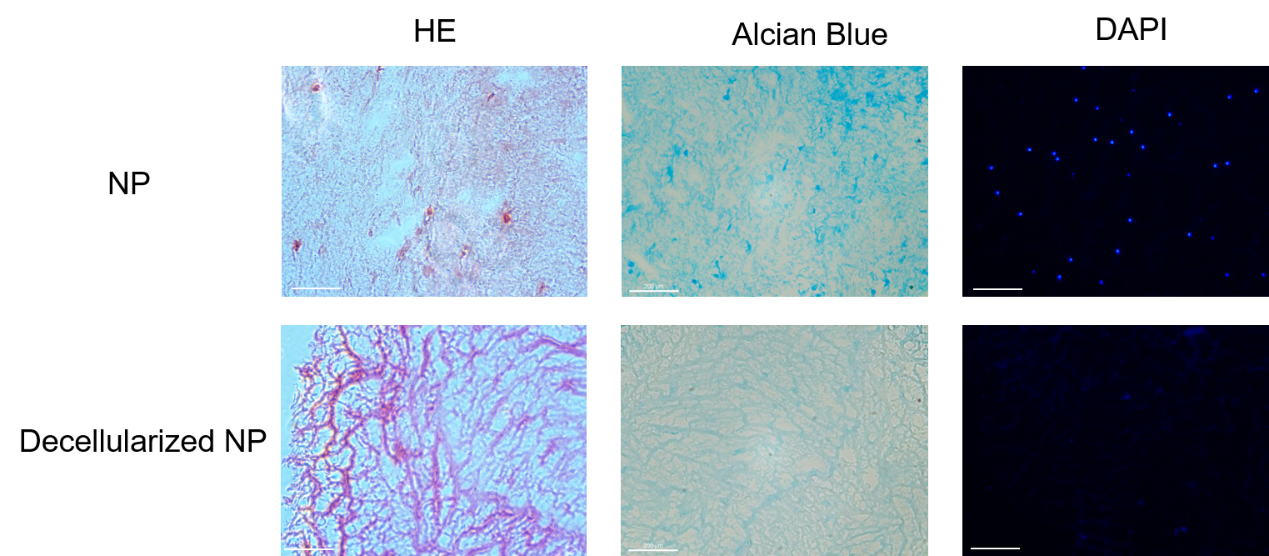


**Supplementary Fig. S3.** H&E staining, Alcian blue staining and DAPI staining of NP tissue and decellularized NP tissue confirmed the absence of nuclei after processing. Scale bar = 200 μm

**Supplementary Table S1.** Sequence used in this study.

|  | Sequences |
| --- | --- |
| Col II | Forward：TGGACGCCATGAAGGTTTTCT  Reverse：TGGGAGCCAGATTGTCATCTC |
| Aggrecan | Forward：GTGCCTATCAGGACAAGGTCT  Reverse：GATGCCTTTCACCACGACTTC |
| NLRP3 | Forward：CGTGAGTCCCATTAAGATGGAGT  Reverse：CTCGACAGTGGATATAGAACAGA |
| MMP13 | Forward：TCCTGATGTGGGTGAATACAATG  Reverse：GCCATCGTGAAGTCTGGTAAAAT |
| Caspase-1 | Forward：TTTCCGCAAGGTTCGATTTTCA  Reverse：GGCATCTGCGCTCTACCATC |
| mIL-1β | Forward：ATGATGGCTTATTACAGTGGCAA  Reverse：GTCGGAGATTCGTAGCTGGA |
| GAPDH | Forward：CAAATTCCATGGCACCGTCA  Reverse：GACTCCACGACGTACTCAGC |
